# Proteasome inhibitor bortezomib enhances the effect of standard chemotherapy in small cell lung cancer

**DOI:** 10.18632/oncotarget.21221

**Published:** 2017-09-23

**Authors:** Sanaz Taromi, Florentine Lewens, Ruza Arsenic, Dagmar Sedding, Jörg Sänger, Almut Kunze, Markus Möbs, Joana Benecke, Helma Freitag, Friederike Christen, Daniel Kaemmerer, Amelie Lupp, Mareike Heilmann, Hedwig Lammert, Claus-Peter Schneider, Karen Richter, Michael Hummel, Britta Siegmund, Meike Burger, Franziska Briest, Patricia Grabowski

**Affiliations:** ^1^ Department of Medicine, Division of Hematology and Oncology, University Medical Center, Freiburg, Germany; ^2^ Department of Gastroenterology, Infectious Diseases, Rheumatology CC13, Charité-Universitätsmedizin, Berlin, Germany; ^3^ Department of Chemistry and Biochemistry, Freie Universität (FU), Berlin, Germany; ^4^ Institute of Pathology, Bad Berka, Germany; ^5^ Institute of Pathology, Charité-Universitätsmedizin, Berlin, Germany; ^6^ Institute of Biology, Humboldt-Universität, Berlin, Germany; ^7^ Department of General and Visceral Surgery, Zentralklinik Bad Berka GmbH, Bad Berka, Germany; ^8^ Institute of Pharmacology and Toxicology, Jena University Hospital, Jena, Germany; ^9^ Department for Oncology, Zentralklinik Bad Berka GmbH, Bad Berka, Germany; ^10^ Department of Gastroenterology and Endocrinology, Zentralklinik Bad Berka GmbH, Bad Berka, Germany; ^11^ Department of Medical Immunology, Charité Universitätsmedizin, Berlin, Germany

**Keywords:** FOXM1, *in vivo*, SCLC, mouse model, lung cancer

## Abstract

Small cell lung cancer (SCLC) is an aggressive cancer showing a very poor prognosis because of metastasis formation at an early stage and acquisition of chemoresistance. One key driver of chemoresistance is the transcription factor Forkhead box protein M1 (FOXM1) that regulates cell cycle proliferation, maintenance of genomic stability, DNA damage response, and cell differentiation in numerous tumor entities. In this study we investigated the role of FOXM1 in SCLC progression and analyzed the effect of FOXM1 inhibition using two proteasome inhibitors, bortezomib and siomycin A. FOXM1 was strongly expressed in patient-derived SCLC samples (n=123) and its nuclear localization was associated with the proliferation marker Ki-67. Both proteasome inhibitors successfully inhibited FOXM1 expression leading to a significantly reduced proliferation and a decreased mitotic rate along with cell cycle arrest and apoptosis induction. These effects were further enhanced by addition of bortezomib to standard chemotherapy. Treatment of mice bearing chemoresistant SCLC xenografts with bortezomib reduced the mean bioluminescence signal of tumors by 54%. Similarly, treatment with cisplatin as a standard chemotherapy reduced the mean bioluminescence signal of tumors by 58%. However, in combination with standard chemotherapy bortezomib further reduced the mean bioluminescence signal by 93% (p=0.0258). In conclusion, we demonstrate the effect of bortezomib in inhibiting FOXM1 expression and thus in sensitizing resistant SCLC cells to standard chemotherapy. Thus, addition of bortezomib to standard chemotherapy might potently improve SCLC therapy, particularly in an extensive cancer stage.

## INTRODUCTION

Lung cancer is still the leading cause of cancer-related death worldwide [1]. Small cell lung cancer (SCLC) accounts for 15-20% of all lung cancer cases and is characterized by an aggressive disease progression [2, 3]. It is often diagnosed at a late stage with frequent metastases [4]. Although SCLC reacts highly sensitive to chemo- and radiotherapy at the initial treatment step, most patients develop a tumor relapse, resulting in a poor median survival period of 9-12 months [5, 6]. The high mortality rate of those patients is due to acquired chemotherapy resistance [7]. Thus, novel therapeutics strategies targeting resistance mechanisms are urgently needed.

In this context, the proteasome inhibitor bortezomib is becoming more and more important due to a remarkable power in re-sensitization of resistant tumors combined with relatively low side effects [8]. Bortezomib (Velcade, formerly PS-341) is a highly selective inhibitor of the 26S proteasome [9]. It is the first proteasome inhibitor approved by the US FDA and EU CPMP for the treatment of patients diagnosed with relapsed/refractory multiple myeloma [10, 11]. By inducing multiple mechanisms, bortezomib displays antiproliferative, proapoptotic, and antiangiogenic properties [12]. Along this line, bortezomib induces NF-kappaB inhibition leading to apoptosis of various cancer cells and sensitizing them to chemotherapy and radiotherapy [13–15]. NF-kappaB is highly overexpressed in SCLC cells and is correlated with the development of chemoresistance [16]. Thus, particularly in addition to standard chemotherapy, a proteasome inhibition may abrogate chemoresistance and maintain a promising outcome in SCLC treatment.

Moreover, bortezomib and other proteasome inhibitors, such as the clinically undocumented but experimentally used proteasome inhibitor siomycin A, were also shown to induce apoptosis of cancer cells by inhibiting the activity and overexpression of Mammalian Forkhead box M1 (FOXM1) [17]. FOXM1 belongs to the forkhead superfamily of transcription factors and as such facilitates cell differentiation, proliferation, invasion, and metastatic dissemination [18]. Multiple downstream targets, such as cyclin A/B, cdc25B, aurora A/B, survivin, and Ki-67 directly determine cell survival and mitosis, whereas the degradation of the CDK inhibitors p27 and p21 attenuates apoptosis [19–24]. Hence, FOXM1 also increases resistance to oxidative stress, DNA damage, and apoptosis [25]. In the context of cell proliferation, FOXM1 drives G1–S and G2–M transition of the cell cycle [21]. Furthermore, FOXM1 has been implicated in angiogenesis by triggering the VEGF-dependent angiogenic switch and in invasion by triggering MMP-2 and MMP-9 release [26–28]. Overexpression of FOXM1 is associated with poor prognosis of lung cancer patients [29–31]. These facts imply that FOXM1 may play a pivotal role in SCLC progression. Whether FOXM1 plays a role in development of chemoresistance in patients and thus may represent an attractive target in SCLC therapy remains unknown. Here we investigated the effect of bortezomib on FOXM1 expression and thus in SCLC therapy. Treatment of chemoresistant SCLC cells with proteasome inhibitors suppressed NF-kappaB and FOXM1 activity, induced cell cycle arrest and apoptosis and in line with this reduced cell proliferation. In a xenograft mouse model treatment with bortezomib potently enhanced the effect of standard chemotherapy. Our findings underscore the potential of proteasome inhibitor bortezomib as re-sensitizing agent in combination with standard therapy.

## RESULTS

### SCLC cells express neuroendocrine markers

**Figure 1:**
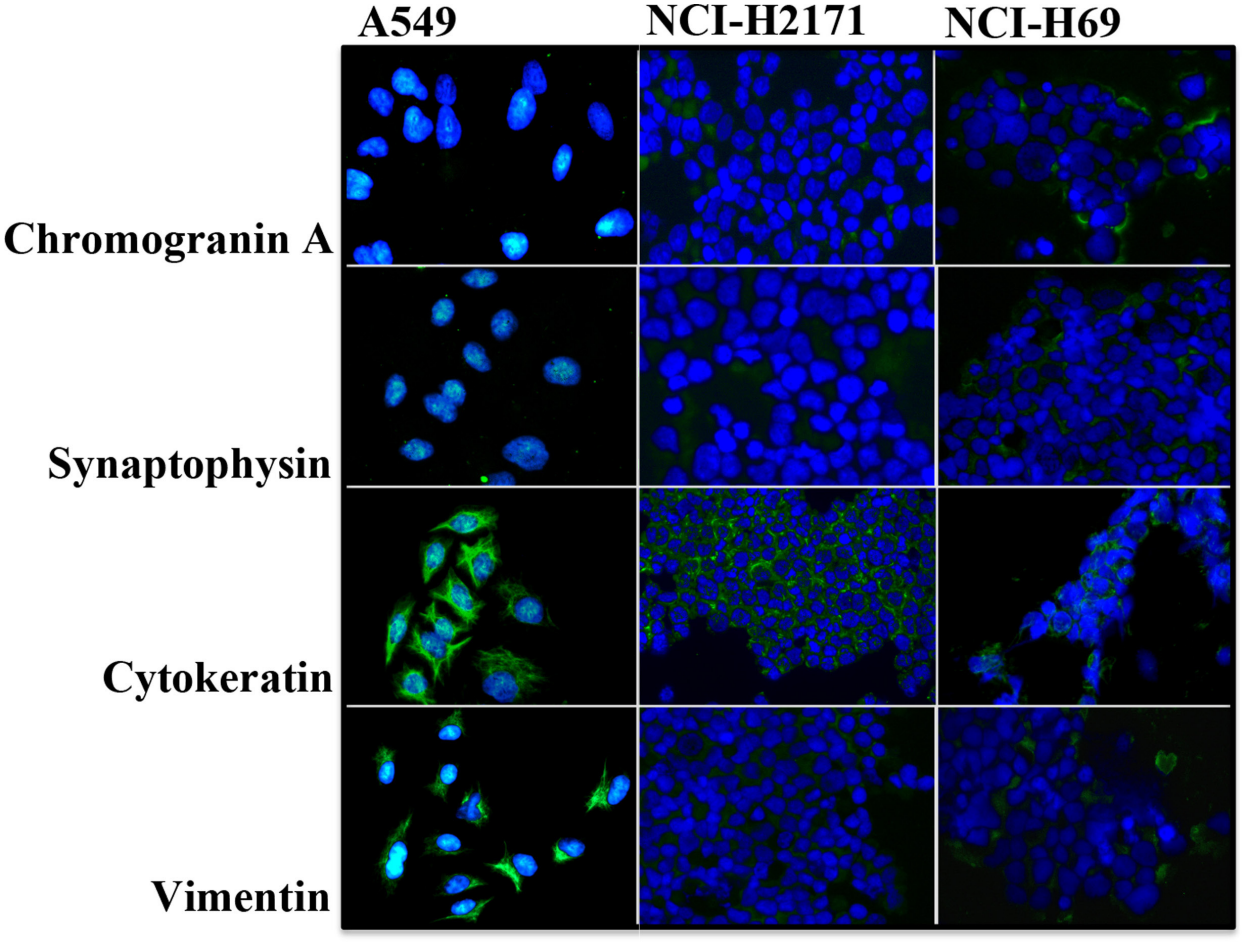
SCLC cells exhibit a characteristic expression of neuroendocrine and differentiation markers Representative immunofluorescence images demonstrate expression of the markers chromogranin (A), synaptophysin, cytokeratin and vimentin by neuroendocrine SCLC cell lines NCI-H2171 and NCI-H69 in comparison to the non-neuroendocrine NSCLC cell line NCI-A549 (200 x magnification).

In order to determine the characteristic features of SCLC cells we analyzed the expression of neuroendocrine and differentiation markers. Both SCLC cell lines NCI-H2171 and NCI-H69 showed expression of the neuroendocrine markers chromogranin A and synaptophysin (Figure 1). In contrast, the non-neuroendocrine NSCLC cell line A549, used as a control, was completely negative for these neuroendocrine markers. Compared to the control cell line, the expression of vimentin was very low in both SCLC cell lines NCI-H2171 and NCI-H69, indicating a less mesenchymal (dedifferentiated) phenotype.

### Nuclear FOXM1 localization in patient-derived SCLC samples is associated with increased Ki-67 expression

**Figure 2:**
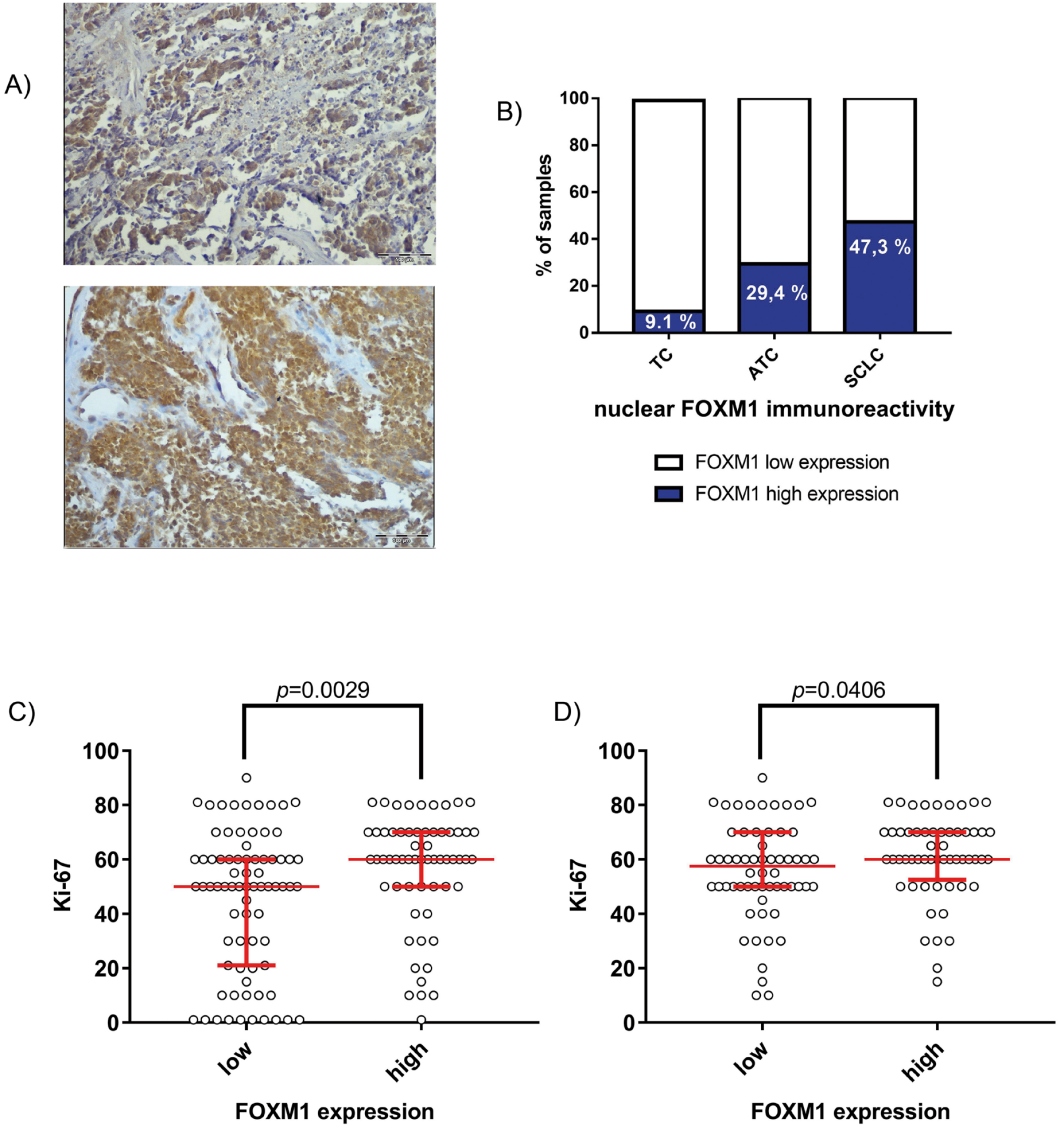
Expression of FOXM1 in patient-derived SCLC samples (**A**) Immunohistochemistry of FOXM1 demonstrated a high percentage of strong nuclear FOXM1 expression in SCLC (58/123; 47.3%). Representative pictures of SCLC show strong nuclear FOXM1 expression (upper panel) or strong immunoreactivity in both, cytoplasm and nucleus (lower panel). Light microscopy, 200x magnification (scale bar=500μm). **(B)** Compared to the well-differentiated carcinoid entities ATC (5/17; 29.4%) and TC (1/11; 9.1%), SCLC samples show enhanced nuclear FOXM1 expression (p=0.026). **(C)** Nuclear FOXM1 expression was associated with high Ki-67 index in all analyzed neuroendocrine lung cancers in general (p=0.0029) and **(D)** in SCLC in particular (p=0.0406).

To prove a clinical relevance of FOXM1 in SCLC progression we analyzed FOXM1 expression in 123 patient-derived SCLC samples performing immunohistochemical staining and compared it to other lung cancer entities. We could demonstrate that FOXM1 was strongly expressed in SCLC (Figure 2). High immunoreactivity for nuclear FOXM1 was detected in 47.3% (58/123) of the SCLC samples, whereas only 29.4% (5/17) of the atypical carcinoid (ATC) samples and 9.1% (1/11) of the typical carcinoid (TC) samples were positively stained for nuclear FOXM1. Thus, nuclear FOXM1 expression was related to the aggressiveness of the neuroendocrine lung cancer entity (p=0.026). Additionally, we demonstrated that the FOXM1 cytosolic localization score was strictly opposite to the nuclear FOXM1 score determined. Concerning cytosolic localization, we found that 69.9% (86/123) of the SCLC samples were positive, whereas 96.4% (27/28) of carcinoid samples were immunoreactive for inactive FOXM1 in the cytoplasm (p=0.004). Cytosolic FOXM1 is thus lower in SCLC than in BP-NET. A cytosolic FOXM1 immunoreactivity does not exclude a nuclear FOXM1 presence in the same case. It only indicates a detectable percentage of inactive (nuclear excluded) FOXM1. Nuclear FOXM1 localization is associated with increased aggressiveness of tumors. Consequently, both scores were related complementary to each another, indicating, that FOXM1 nuclear shuttling rather than total FOXM1 expression determines its biological activity.

Most notably, FOXM1 nuclear immunoreactivity was strongly associated with the clinical proliferation marker Ki-67. This effect was significant throughout all analyzed lung NET entities (p=0.0029) as well as for SCLC in particular (p=0.0406).

### Treatment with proteasome inhibitors suppresses proliferation of SCLC cells

**Figure 3:**
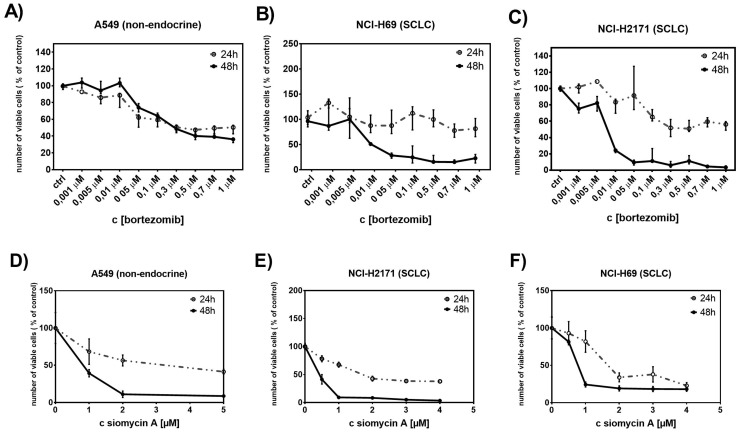
Treatment with bortezomib or siomycin A suppresses SCLC cell proliferation The antiproliferative effect of bortezomib (0.001 μM to 1μM) or siomycin A (0.1μM to 5μM) on SCLC cells was quantified using WST-1 viability assay. Both, bortozomib and siomycin A treatments (for 24h and 48h) reduced the number of viable SCLC cells in a time and concentration-dependent manner **(B, C, E, F)**. The effect of bortezomib and siomycin A on the control A549 cells was less effective **(A, D)**.

In order to identify the role of FOXM1 in SCLC progression we analyzed the effect of the proteasome inhibitors bortezomib and siomycin A on cell proliferation of SCLC. Both inhibitors are known to potently suppress expression of FOXM1 [17]. The cells were treated with different concentrations of bortezomib (ranging between 0.001 μM and 1 μM) and siomycin A (ranging between 0.1 μM and 5 μM) for 24 h and 48 h. The proliferation rate of treated cells was quantified using WST-1 viability assay. Previously, the IC50 values of both inhibitors were determined to persist within the nanomolar range (Supplementary Figure 1 and 2). Both SCLC cell lines showed strong sensitivity to FOXM1 inhibition (Figure 3). Bortezomib suppressed cell proliferation in a time- and concentration-dependent manner. Compared to the control cell line A549, bortezomib treatment achieved up to 46-fold higher efficiency in SCLC cells. Already low concentrations of bortezomib (0.05 μM) showed 80-90% reduction in cell viability in SCLC cells. Even the prolonged treatment of control A549 cells (72 h) showed no improvement in bortezomib efficiency. Suppression of SCLC cell viability upon siomycin A treatment was similar to bortezomib treatment. Already low concentrations of siomycin A (0.5 μM) reduced cell viability of SCLC cells to <50%. The viability of the control A549 cells was reduced to ∼70%. Nevertheless, on control A549 cells siomycin A showed an increased inhibitory effect than bortezomib.

### Treatment with proteasome inhibitors induces cell cycle arrest and apoptosis of SCLC cells

**Figure 4:**
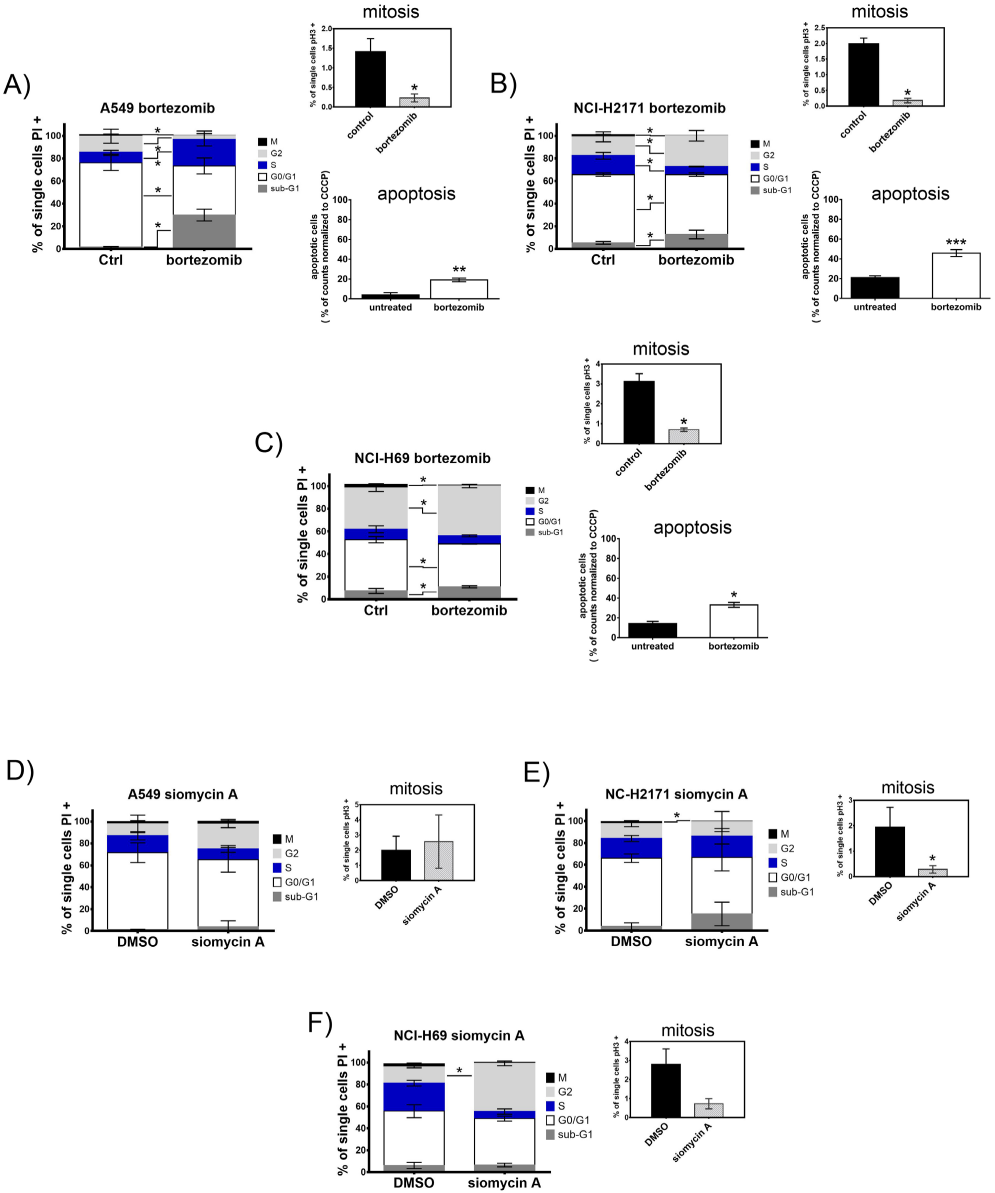
Treatment with bortezomib or siomycin A induces cell cycle arrest and apoptosis of SCLC cells SCLC cell lines NCI-H69 and NCI-H2171 were treated with bortezomib (0.05 μM; 0.5 μM) or siomycin A (0.8 μM; 0.15 μM) and compared to the control A549 cells treated with 1.5 μM bortezomib or 1 μM siomycin A. Cell cycle stages are shown as percentage of cells compared to all analyzed cells. Mitotic index was analyzed separately by phospho-histone H3 immunoreactivity and displayed as percentage of mitotic cells in respect to all analyzed cells. **(A-F)** Bortezomib as well as siomycin A induced cell cycle arrest at G2-M transition and apoptosis in SCLC cells after 48h. Treatment with both inhibitors decreased the M-population and increased the S-population in SCLC cells (representative graphs out of three independent experiments). Apoptosis was measured by JC-1 mitochondrial depolarization assay after 20h of treatment. All cell lines significantly induced apoptosis upon bortezomib treatment. ^*^p<0.05; ^**^p<0.01; ^***^p<0.001; ^****^p<0.0001.

In order to clarify the effect of proteasome inhibitors on cell proliferation, we analyzed the cell cycle of treated cells using a mitotic index flow cytometry analysis. The cells were treated for 48h with bortezomib or siomycin A. In all cell lines treatment with bortezomib or siomycin A significantly reduced the mitotic cell population detected by phosphorylated histone H3 (pH3) staining (Figure 4). In accordance to the cell cycle arrest at the G2-M transition state both inhibitors significantly increased G2-phase population in SCLC cells. Compared to the control A549 cells, bortezomib decreased S-phase population in both SCLC cell lines (A-C). Treatment with siomycin A decreased S-phase only in NCI-H69 cells (F). In addition to cell cycle arrest, bortezomib induced apoptosis in all cell lines (right lower graphs (A-C)).

### Bortezomib enhances the effect of cisplatin-induced cell cycle arrest and apoptosis of SCLC cells

**Figure 5:**
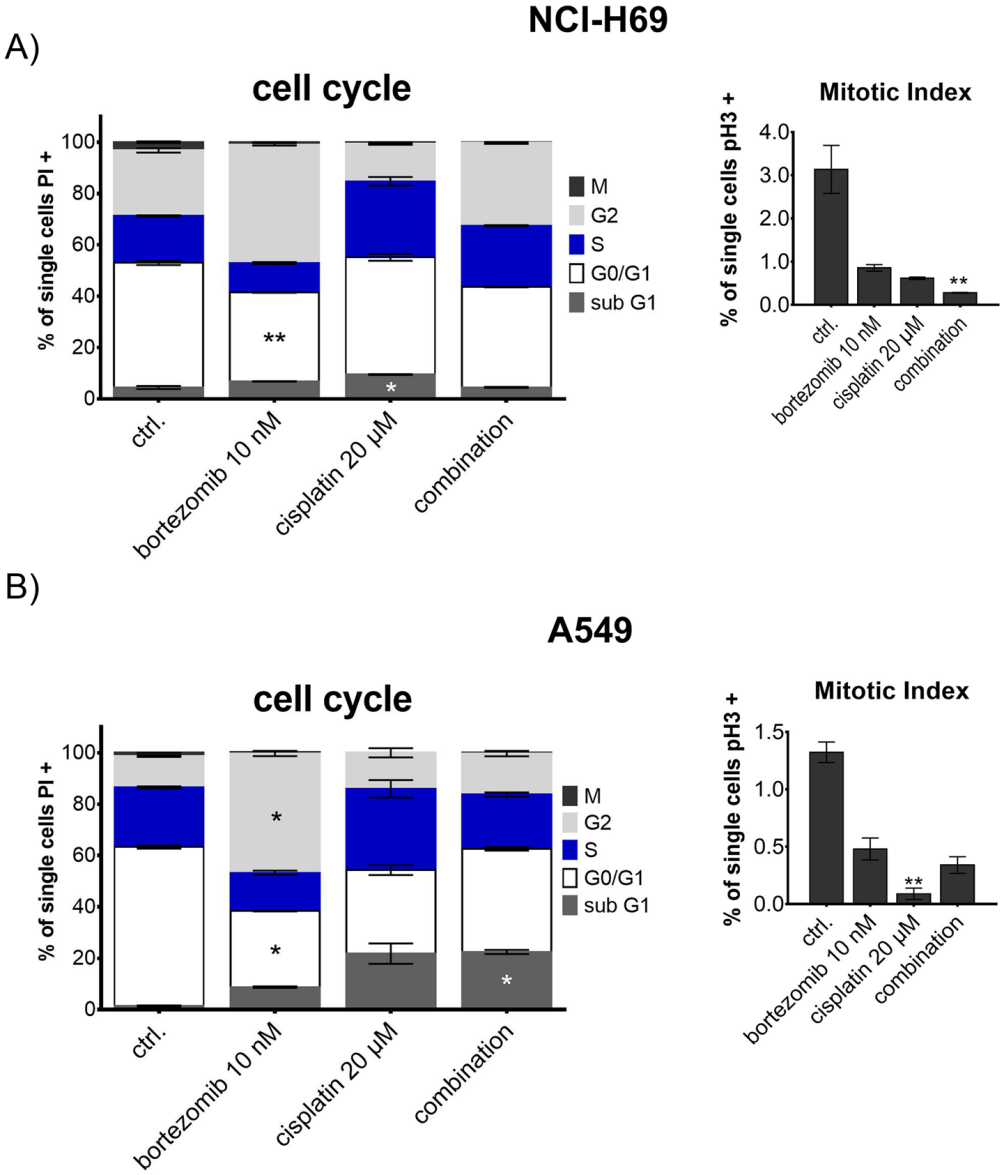
Cell cycle analysis of NCI-H69 and A549 after treatment with bortezomib and cisplatin Cells were treated with 10 nM (NCI-H69) and 100 nM (A549) bortezomib, 20 μM cisplatin, or a combination of both substances for 48h. Cell cycle stages are shown as percentage of cells compared to all analyzed cells. Mitotic index was analyzed separately by phospho-histone H3 immunoreactivity and displayed as percentage of mitotic cells in respect to all analyzed cells. Treatment of NCI-H69 cells induced predominantly G2/M arrest, resulting in a significant reduction of mitotic cells after combined treatment. In contrast, the control cell line A549 displayed a stronger apoptotic response, but the mitotic cell population only decreased significantly in the cisplatin monotherapy treatment group. ^*^p<0.05; ^**^p<0.01; ^***^p<0.001; ^****^p<0.0001.

To further identify the effect of proteasome inhibitors on chemosensitivity of SCLC cells we analyzed the cell cycle feature of NCI-H69 cells treated with the combination of bortezomib and cisplatin. We concentrated on NCI-H69 cells as they were established from the human chemoresistant SCLC metastasis and thus represent the chemoresistant SCLC nature. Cells were treated with 10 nM (NCI-H69) and 100 nM (A549) bortezomib, 20 μM cisplatin, or a combination of both substances. Treatment of NCI-H69 cells induced predominantly G2/M arrest, resulting in a significant reduction of mitotic cells after combined treatment. Compared to cisplatin monotherapy, addition of bortezomib further enhanced G2/M arrest resulting in a significant reduction of mitotic cell population and increased G2 population (Figure 5). In contrast, the control cell line A549 displayed a stronger apoptotic response (indicated by sub G1), but the mitotic cell population only decreased significantly in the cisplatin monotherapy treatment group.

### Treatment of SCLC cells with bortezomib affects FOXM1 signaling pathway at several levels

**Figure 6:**
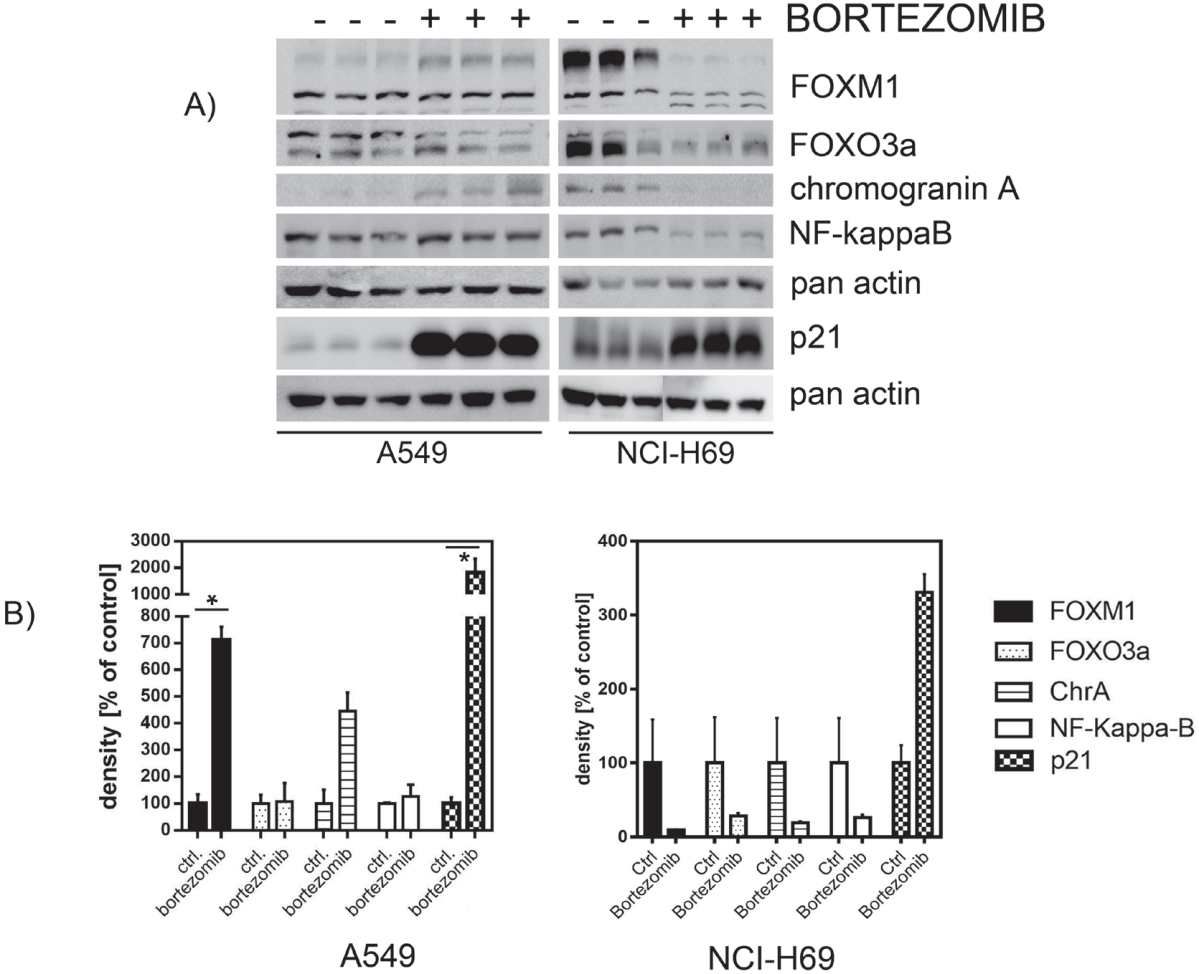
Western blot analysis revealed affected protein expression after treatment with bortezomib SCLC cell line NCI-H69 and the A549 control cell line were treated with bortezomib for 48h (individual IC50). The levels of signaling partners FOXM1, FOXO3a, chromogranin A, p21, and NF-kaB were determined by immunoblotting and densitometrically compared to the untreated control. Treatment with bortezomib reduced the abundance of FOXM1, FOXO3a, chromogranin A, and NF-kB, but strongly enhanced p21 expression (unless not significant). Blots were cropped to improve the conciseness of the presentation.

In order to identify the inhibitory mechanisms of bortezomib on chemoresistant SCLC cells we analyzed the expression of FOXM1 signaling partners in control and SCLC cells. Both, A549 and NCI-H69 cells were treated with bortezomib for 48 h and analyzed by immunoblotting for expression of FOXM1, FOXO3a, chromogranin A, NF-kappaB and p21-protein. FOXM1 and FOXO3a are known as antagonizing key players in cancer progression, proliferation and drug resistance [32]. Teatment with bortezomib showed no effects in control A549 cells but potently inhibited expression of both, FOXM1 and FOXO3a in SCLC cells (Figure 6). Similarly, bortezomib treatment reduced expression of the neuroendocrine marker chromogranin A and of the proliferation regulator NF-kappaB in SCLC. Moreover, treatment with bortezomib strongly enhanced expression of cyclin-dependent kinase inhibitor p21 that potently blocks cell-cycle progression in different cancer entities.

### Bortezomib in combination with cisplatin affects gene expression of NCI-H69 cells

**Figure 7:**
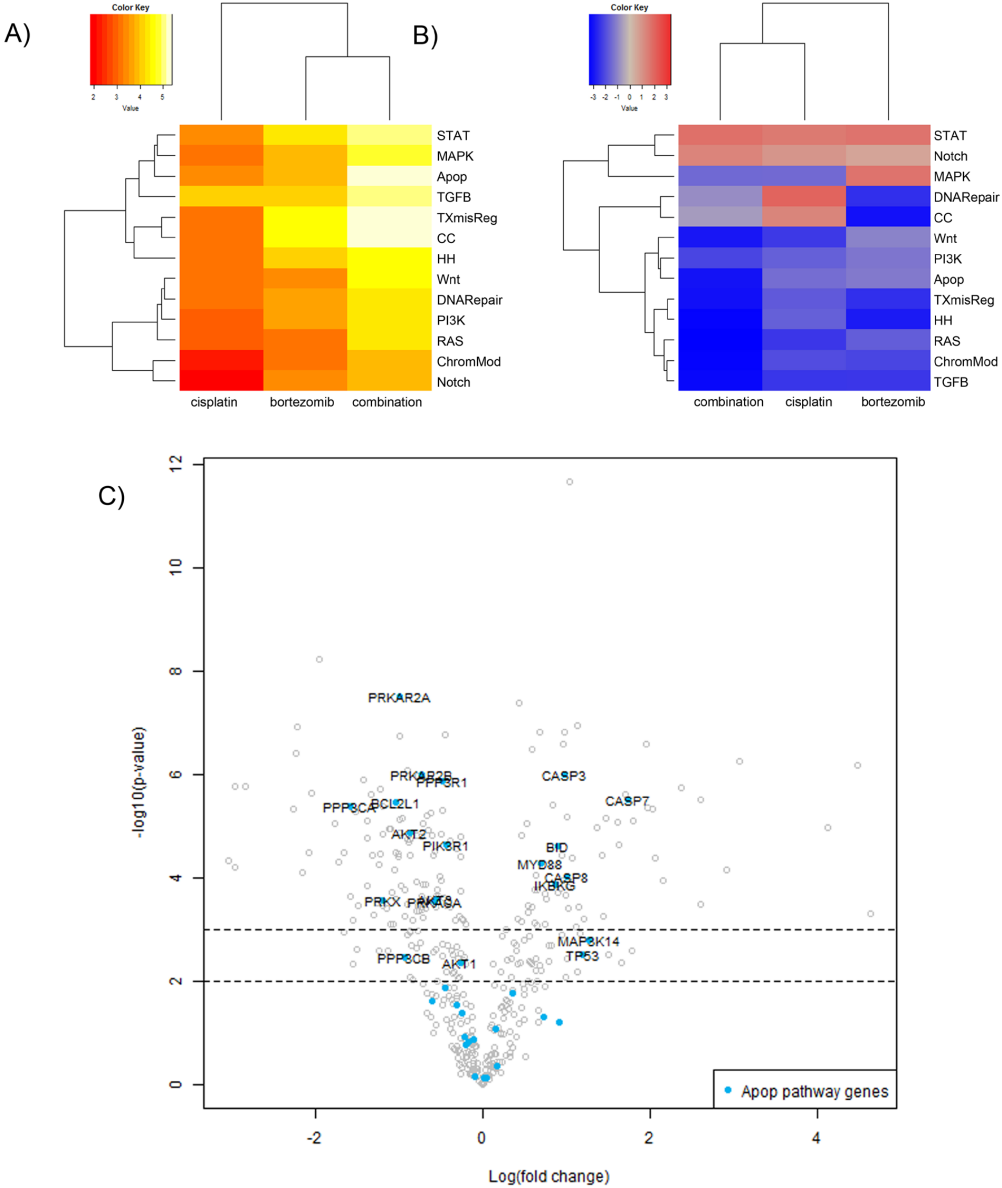
Gene expression analysis of NCI-H69 cells after treatment with cisplatin or bortezomib *in vitro* NCI-H69 cells were treated with 50 nM bortezomib, 10 μM Cisplatin or a combination for 48h. The isolated mRNA was analyzed by the nCounter^®^ PanCancer multiplex gene expression array (Nanostring^®^ technologies). **(A)** The heatmap displays the global significance scores of pathways after treatment and indicates that apoptosis, transcriptional pathways and cell cycle regulation were the most affected mechanisms. **(B)** Directed global significance statistics measure the extent to which a pathway’s genes are up- or downregulated after treatment. Red denotes pathways whose genes exhibit extensive overexpression with the covariate, blue denotes pathways with extensive underexpression. The blue heatmaps denote that the gene expression is predominantly affected in a negative way after combined treatment. **(C)** The volcano plot demonstrates the functional specification: whereas mainly pro-apoptotic genes are upregulated, antiapoptotic genes are downregulated. Apoptosis is therefore induced, irrespective from the overall negative pathway score. Associated Pathways: STAT, PI3K, RAS, MAPK, Wnt, Notch, TGFB, DNA repair, Apop=apoptosis, TXmisReg=transcriptional misregulation, CC=cell cycle, TGFB=TGF-beta, ChromMod= chromatin remodeling, HH=Hedgehog.

To further identify the inhibitory mechanisms of bortezomib at the mRNA expression level, we analyzed the changes in gene expression of treated SCLC cells. NCI-H69 cells were treated with 50 nM bortezomib, 10 μM Cisplatin or a combination for 48h. The isolated mRNA was analyzed by the nCounter^®^ PanCancer multiplex gene expression array (Nanostring^®^ technologies). In bortezomib or cisplatin treated cells, apoptosis, transcription and cell cycle regulation were the most affected mechanisms (Figure 7A). Combination of bortezomib and cisplatin further enhanced this effect. Overall, the gene expression was predominantly affected in a negative way after combined treatment (Figure 7B). Whereas mainly proapoptotic genes were upregulated, antiapoptotic genes were downregulated (Figure 7C). Apoptosis was therefore induced, irrespective from the overall negative pathway score. The more detailed PANTHER pathway overrepresentation analysis (supplementary Figure 3C), demonstrates a strong overrepresentation (>15-fold) of genes involved in the p53 associated pathways as well as an overrepresentation of genes that regulate apoptosis and FAS signaling. The death receptor pathway of apoptosis induction, downstream of FAS is found affected only after combined treatment.

To further explore the distinct role of FOXM1 in the cellular response to bortezomib, we performed a siRNA-mediated knockdown of FOXM1 and analyzed the gene expression pattern by a subsequent nCounter^®^ array. FOXM1 knockdown resulted in reduced expression of SKP2, which is involved in the regulation of p21 and p27 [21] and in upregulated STAT1 expression, which is necessary for FAS induced apoptosis [33] (Supplementary Figure 4). Gene expression raw data can be found in Supplementary Tables 1-4.

### Combination of bortezomib and cisplatin potently suppresses the growth of SCLC tumors *in vivo*

**Figure 8:**
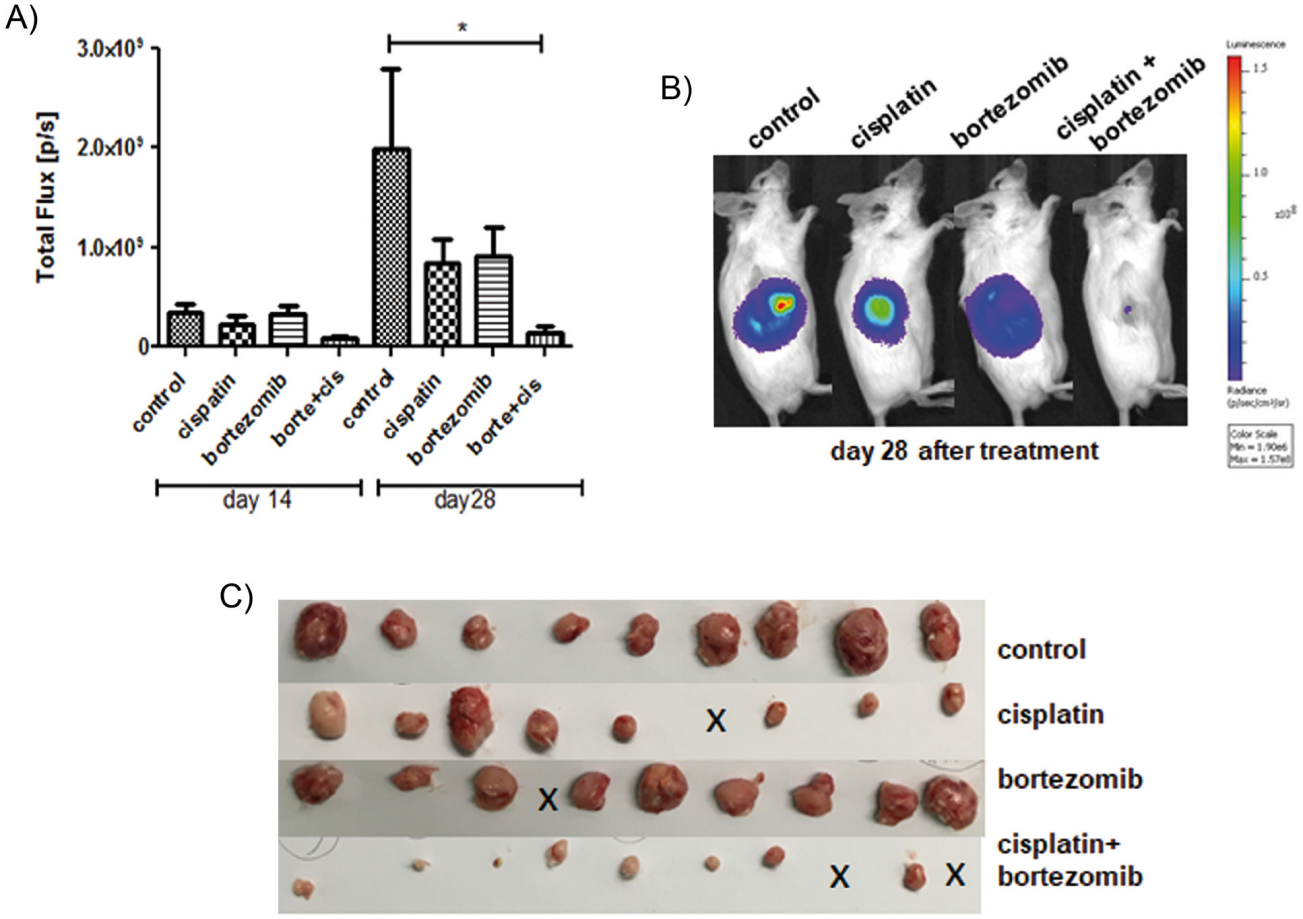
Addition of bortezomib enhances the antiproliferative effect of cisplatin *in vivo* Tumor-bearing mice were treated with bortezomib (n=10) or cisplatin (n=9) as a monotherapy, or with a combination of both therapeutics (n=10) and compared to solvent treated controls (n=9). Cisplatin (5 mg/kg/day) was administered by intraperitoneal injection on days 14 and 21 following tumor injection. Bortezomib (1 mg/kg/day) was administered by intraperitoneal injection on days 14, 18, 21 and 25 following tumor injection. Cisplatin reduced the mean tumor BL signal (total flux p/s) by 58% and bortezomib reduced the mean tumor BL signal by 54%, respectively. Combination of both therapeutics reduced the mean BL signal by 93% of the control tumor (^*^ = p < 0.05).

In the next step, we analyzed whether addition of bortezomib might improve the efficacy of standard chemotherapy. H69-Luc-GFP cells were injected subcutaneously into the right flanks of immunodeficient Rag2^-/-^γc^-/-^ mice. Highly sensitive BL imaging was applied immediately after tumor injection enabling detection of very small non-palpable tumors. Tumor growth was then monitored using BLI on day 14 and day 28 after tumor injection. Treatment of mice started two weeks after tumor inoculation, at the timepoint where the palpable tumors were established. Tumor-bearing mice were randomized into four groups and treated with bortezomib or cisplatin as a monotherapry, or with a combination of both. Bortezomib was administered by intraperitoneal injection at a concentration of 1 mg/kg/day on days 14, 18, 21 and 25 after tumor inoculation. Cisplatin was administered by intraperitoneal injection at a concentration of 5 mg/kg/day on days 14 and 21 after tumor inoculation. In comparison to the control group, cisplatin or bortezomib as a monotherapy reduced the growth of the SCLC tumors by 58% or 54%, respectively (Figure 8). Combination of both therapeutics reduced the growth of tumors by 93% of the control volume (^*^ = p < 0.05). Two out of 10 tumors were completely regressed in a combination treated group (Figure 8C, indicated by x).

## DISCUSSION

The current SCLC treatment remains limited to the combined chemotherapy of cisplatin/carboplatin and etoposide, which results in an unsatisfactory median survival time of 8 to 13 months [34–36]. Targeted therapy specifically developed for this tumor entity is still lacking. In order to improve the current SCLC therapy by targeting chemoresistance mechanisms we focused on the role of FOXM1 in SCLC progression. This study emphasizes FOXM1 as a crucial oncogene-like marker, which can be effectively targeted by the proteasome inhibitor bortezomib. Bortezomib as well as siomycin A successfully inhibited FOXM1 expression leading to a significantly reduced proliferation, decreased mitotic rate, cell cycle arrest and apoptosis. Treatment with cisplatin as a standard chemotherapy reduced the mean bioluminscence signal of tumors by 58%. Similar effects showed bortezomib treatment by reducing the mean bioluminescence signal of tumors by 54%. Importantly, the combination of bortezomib and cisplatin potently reduced the mean bioluminescence signal by 93%.

In recent years, the significance of FOXM1 expression has continuously gained growing attention as one of the driving factors in cancer progression [37]. It has been shown to support tumor cell survival and epithelial-mesenchymal transition in ovarian carcinoma cells [38]. In breast cancer cells FOXM1 expression conferred cisplatin resistance [39]. Particularly, the nuclear localization of FOXM1 was associated with acquired chemoresistance in tumors [40]. The role of FOXM1 in neuroendocrine pulmonary tumors has been recently assessed in a small study that failed to demonstrate a clear function of FOXM1 as tumor marker and proposed a combined FOXM1/p21 and FOXM1/p27 score [41]. In our study, immunohistochemistry in 151 cases of ratified BP-NETs demonstrated that FOXM1 as single marker was strongly upregulated in SCLC samples compared to the better-differentiated carcinoid tumors controls. High immunoreactivity for nuclear FOXM1 was detected in 47.3% (58/123) of the SCLC samples. Most notably, FOXM1 nuclear immunoreactivity was strongly associated with the clinical proliferation marker Ki-67. This effect has been demonstrated for gastroenteropancreatic neuroendocrine tumors as well [42]. In contrast to intestinal neuroendocrine tumors, Ki-67 is not a clinically established tumor marker in neuroendocrine lung tumors. Nevertheless, recent publications demonstrate that Ki-67 labeling index has a high level of specificity to distinguish TC and ATC from LCNEC and SCLC and is proposed for clinical classification guidelines [43–45].

Unless this study is limited due to the small number of control BP-NET, the high expression of FOXM1 in SCLC related to BP-NET could be significantly demonstrated.

In accordance with the pivotal role of FOXM1 in cancer, its inhibition suppressed cell proliferation and tumor growth of breast cancer and promoted the cytotoxic effects of platinum compounds, doxorubicin hydrochloride and olaparib in epithelial ovarian cancer [46, 47]. The proteasome inhibitors bortezomib and siomycin A have been shown to potently inhibit FOXM1 expression [17, 48]. In this study human SCLC cell lines responded to proteasome inhibitor treatment by down-regulation of FOXM1, cell cycle arrest, and apoptosis induction, even under low-dose treatment. Following proteasome inhibition, the expression of FOXM1 as well as of several common p53 and FOXM1 target proteins were affected in a classical SCLC cell line NCI-H69. Using western blot analysis, we demonstrated that the CDK inhibitor and tumor suppressor protein p21 was upregulated. This cell cycle regulator is downregulated by FOXM1 [23] and often mutually upregulated by p53 [49]. Bortezomib affects the P53/RB/P21-dependent stress response cascades and mitotic protein expression, at least in part by down-regulation of FOXM1. As p21 and E2F are further under the transcriptional control of FOXM1 [24] and the effect of proteasome inhibition seems to be independent from the mutational status of p53, FOXM1 seems to be a mediating target of proteasome inhibitor response. As bortezomib is a proteasome inhibitor and not explicit to FOXM1 inhibition, it is not possible to conclude specific mechanistic connections. Nevertheless, analyzing different proteasome inhibitors, Bhat *et al.* showed that the negative regulation of FOXM1 is a general mechanism of these drugs and might drive their anticancer effect [17]. Gene expression analyses revealed that knockdown of FOXM1 reduced the expression of the p21 regulator SKP2 and induced proapoptotic STAT1. Nevertheless, the clear role of FOXM1 in mediating the response to bortezomib treatment remains to be further investigated.

Western blot analysis showed also a decrease of NF-kappaB p65 and FOXO3a. The tumor suppressor FOXO3a is associated with chemoresistance in breast cancer [50]. The reduction of FOXO3a might indicate a high PI3K or MAPK-pathway activation, as AKT and ERK1/2 are known to phosphorylate FOXO3a, thus, triggering its degradation. A recent study has demonstrated that the presence of active AKT and subsequently deactivated FOXO3a, in addition to active RB, is capable of determining the quiescence-senescence switch and thus, determining the persistence of a cellular proliferation arrest [51]. NF-kappaB p65 is associated with cell survival and represses essential cell cycle effectors regulated by FOXM1 in other cancers [20, 52]. The important role of NF-kappaB in lung cancer progression has been discussed deeply by Chen *et al.*, who link the enhanced NF-kappaB signaling in lung cancer to chemo- and radiotherapy resistance, thus, highlighting the significance of targeting the NF-kappaB signaling pathway in lung cancer treatment [16]. Inhibition of NF-kappaB signaling pathway by bortezomib sensitized resistant renal carcinoma cells to direct necrotic death [53].

In our study, we could demonstrate the strong chemosensitizing effect of bortezomib in SCLC *in vivo* for the first time. In previously established SCLC xenograft mouse model [54] treatment with the combination of bortezomib and cisplatin showed a total remission of 20% of the tumors. Although bortezomib or cisplatin as monotherapies reduced the mean bioluminscence signal of tumors by 54-58%, the combination of both potently reduced the mean bioluminescence signal by 93%. These findings are consistent with previous studies on neuroblastoma and prostate cancer demonstrating the efficacy of bortezomib in overcoming chemoresistance [55, 56]. Suppression of tumor growth upon bortezomib monotherapy might result from the reduced expression of anti-apoptotic BCL-2, as has been previously shown *in vitro* for SCLC cells [57]. Nevertheless, in early clinical studies bortezomib failed to show single agent activity in SCLC [58]. The reason for the low monotherapeutic efficiency of bortezomib might be the lack of a strong pro-apoptotic trigger in the context of a reduced apoptotic capacity due to several tumor suppressor gene mutations (*TP53, RB*). Therefore, the authors propose to add an apoptotic trigger, such as chemotherapy. Indeed, bortezomib has shown to induce apoptosis combined with chemotherapy in NSCLC [59].

In NSCLC cells bortezomib abrogated chemoresistance by increasing p21 expression and targeting FOXM1 [59, 60]. Moreover, the chemosensitizing effect of bortezomib to platin-based chemotherapy has been recently discussed for NSCLC therapy [61, 62]. In conclusion, our data demonstrate that bortezomib augments the therapeutic efficacy of the standard SCLC chemotherapy. Thus, integration of bortezomib into the standard SCLC therapy may ultimately improve the outcome of patients and be of high value in a wide range of FOXM1-driven cancers.

## MATERIALS AND METHODS

### Patients and samples

In this study, we examined 151 formalin-fixed tissue samples collected by the Department of General and Visceral Surgery at the Zentralklinik Bad Berka GmbH between 2007 and 2012. These specimens included small-cell lung cancer (N=123), atypical carcinoid (N=17), and typical carcinoid (N=11) tumor samples (Table 1). Immunohistochemical characterization and analysis was done blinded and independently by the author P.G. and two experienced pathologists from the Zentralklinik Bad Berka GmbH and the Charité university hospital in Berlin (R.A., J.S.). The inter-observer variability was well below 100% and was discussed until a consensus formed between the researchers. Cases histologically verified as BP-NETs were characterized based on WHO classification and were re-evaluated by morphological criteria. Tumor samples selected for this study exceeded 80% tumor cells each.

**Table 1:** Clinicohistopathological data of tumor biopsies

BP-NETs:					
N=151					
entity		SCLC	ATC	TC	total
**slides (N)**		**123**	**17**	**11**	**151**
**gender**	**male**	88	11	4	103
	**female**	35	6	7	48
**grading**	**G1**	0	3	11	14
	**G2**	0	12	0	12
	**G3**	123	2	0	125
**age (diagnosis)**	**min**	22	25	52	
	**max**	90	76	81	
	**mean**	66,26	59,42	63,66	
N=151					
**slides (N)**		**123**	**17**	**11**	**151**
**tumor staging (T)**	**T≤2**	31	9	8	48
	**T≥2**	80	0	0	80
	**T n/a**	12	8	3	23
N=151					
**slides (N)**		**123**	**17**	**11**	**151**
**lymph node status (N)**	**N= 0**	17	11	10	38
	**N>0**	92	0	0	92
	**N n/a**	14	6	1	21
N=151					
**slides (N)**		**123**	**17**	**11**	**151**
**metastasis status (M)**	**M=0**	48	5	8	61
	**M>0**	68	5	1	74
	**M n/a**	7	7	2	16
N=151					
**slides (N)**		**123**	**17**	**11**	**151**
**Ki67 status**	**Ki67< 2**	0	0	11	11
	**Ki67≥ 2**	123	17	0	140

The table summarizes 151 FFPE tumor samples used for histopathological staining, sub-grouped into the entities small-cell lung cancer (SCLC), atypical carcinoid (ATC), and typical carcinoid (TC).

### Cell culture

Neuroendocrine lung cell lines used in our experiments were grown in pre-coated flasks (Falcon^®^ Tissue Culture Flasks, Sterile, Corning^®^). Small cell lung cancer cell lines were donated by the lab group Burger from Freiburg (NCI-H69) and A. Nonnenmacher (Charité, CBF, Berlin, Germany) (NCI-H2171). Non-neuroendocrine control lung cell line A549 was kindly gifted by A. Weinhäuser (Charité, CBF, Berlin, Germany) and used if indicated. All cell lines were grown in a cell-specific medium supplemented with HEPES, 1% of penicillin-streptomycin, and 10% fetal bovine serum (FBS gold). A549, NCI-H2171, and NCI-H69 were maintained in RPMI-1640 with stable L-glutamine. All cell lines were grown in a humidified atmosphere of 5% CO2 and 95% air, and were cultured less than 15 passages following reception. All cell lines were authenticated by the DSMZ (Braunschweig, Germany in 2013 and 2014) by STR typing. To guarantee neuroendocrine activity, we used immunofluorescence microscopy to regularly test for cell line-specific expressions of neuroendocrine and differentiation markers, such as chromogranin A, synaptophysin, cytokeratin, vimentin, and syntaxin.

### Generation of luciferase-expressing NCI-H69 cells

SCLC cells were transfected with two retroviral transfer vectors, pLib Luci-Neo (LN) and pLib EF1 a Luci-Neo (ELN) as previously described [63]. Transduced cells (H69-Luc-GFP) were selected and cultured in the presence of 500 g/ml of G418 (Life Technologies GmbH, Darmstadt).

### Immunofluorescence

Cells were seeded and grown for 48 hours on cover slips and then fixed in 1:1 acetone/methanol. Next, unspecific bindings were blocked with milk buffer (2%) for 30 minutes and washed in PBS (0.1% BSA and 0.5% triton X). Primary antibody solutions were added to the cells and incubated for one hour at room temperature in a wet chamber. The primary antibodies used were chromogranin A (Progen Biotechnik GmbH, Heidelberg, Germany; 1:10), synaptophysin (Biogenex, CA, USA; 1:10), cytokeratin pan (Biomedical Ag-Dianova Aliquots; 1:200), and vimentin (-)(clone V9, Merck Millipore, Billerica, MA, USA). After incubation, cells were washed in PBS (0.1% BSA and 0.5% triton X) and secondary antibodies were applied (1:200, Alexa Fluor 488 goat anti-mouse and Alexa Fluor 594 goat anti-rabbit; Life Technologies) for two hours in the dark in a wet chamber. After incubation, cells were washed in PBS and fixed in 96% ethanol for two minutes. Slides were dried after approximately 10 minutes and embedded in Roti^®^-Mount FluorCare DAPI (Carl Roth GmbH, Karlsruhe, Germany).

### Immunochemistry

Immunohistochemistry of paraffin-embedded specimens was performed using the peroxidase-anti-peroxidase method previously described in Grabowski *et al.* [54]. The FOXM1 (FOXM1 C-20) antibody was obtained from Santa Cruz Inc. and applied in a 2 μl/ml dilution. The scoring was performed as follows: nuclear staining intensity was determined as negative (0), weak (1), and strong (2), and multiplied by the percentage of the positive cells determined as 0 % (0), ≤10 (1), 11-50 (2) and >51 (3). The resulting score was considered low if <4 and high if ≥4.

The cytosolic FOXM1 score was assessed by staining as 0 (no), 1 (weak), 2 (moderate), or 3 (strong) immunoreactivity. To dichotomize this variable, only moderate and high staining were considered as positive staining. Immunohistochemical evaluation of all slides was done independently by three experts (R.A., J.S., P.G.); among them two experienced pathologists (R.A., J.S.).

### Cell proliferation assay

Cells were seeded 5,000 to 10,000 cells per well in 96-well plates. All empty wells were filled with sterile PBS solution to minimize evaporation effects. Cells were grown in their regular medium for 24 hours before being treated for 24, 48, and 72/96 hours respectively with bortezomib and siomycin A (derived from streptomyces sioyaensis, Sigma-Aldrich, solved in DMSO). The ready-to-use bortezomib solution was provided by the dispensary of the Charité (1 mg/ml solution from Velcade 3.5 mg, Millennium Pharmaceuticals, Inc., Cambridge, MA, USA). For each concentration, we used five wells on each plate. After incubation, the used treatment medium was removed and exchanged with 100 μl of regular medium. Cell proliferation reagent WST-1 (Roche, Applied Science, Penzberg, Upper Bavaria, Germany) was added to each well and carefully re-suspended. Cells were incubated for two hours in the dark at 37°C. Colorimetric analysis was performed in a microplate reader (TECAN Sunrise™, Tecan Group AG, Männedorf, Switzerland) at 450 nm. All experiments were repeated in three individual trials. For statistic evaluation, we used Prism 6 software (GraphPad Software, Inc.).

### Western blot analysis

Following a 48-hour treatment, cells were harvested from culture and lysed. Protein concentrations were determined using Quick Start Bradford Protein assay (Bio-Rad Laboratories, Inc.). SDS Page western blot was performed using a standard protocol with electroblotting onto PVDF membranes and documented by Coomassie staining (Coomassie Brilliant Blue, AppliChem GmbH, Darmstadt, Germany). Primary antibodies were purchased from Cell Signaling Technology Inc. Danvers, MA, USA: pan actin (D18C11), FOXO3a (75D8), NF-kappaB p65; Santa Cruz Biotechnology Inc. Dallas, Texas, USA: FOXM1 (C-20), p21; GeneTex Inc. Irvine, CA, USA: GAPDH; and Progen GmbH Heidelberg: chromogranin A (clone LK2H10). Appropriate horseradish peroxidase (HPR)-conjugated secondary antibodies (Dako Deutschland GmbH, Hamburg, Germany) were subsequently applied after washing and incubated for 1.5 hours. Chemiluminescence was visualized using an enhanced chemiluminescence reaction system (ECL™ prime Western Blotting detection reagent; Amersham™ GE healthcare) and imaged by a Fujifilm LAS-4000 luminescent image reader. Densitometric evaluations of detected chemiluminescence signals were performed by Multigauge V3.1 analysis software. The expression values of each protein was normalized to an internal control in three independent experiments and statistically assessed with Prism 6 software (GraphPad Software, Inc.). For re-probing, membranes were incubated in an acidic glycine buffer.

### Cell cycle analysis

All cell lines were seeded in pre-coated six-well plates for 48 hours. Cells were treated with siomycin A or bortezomib for a further 48 hours in three individual experiments *in vitro*. Treated cells were then harvested, solubilized, and washed in cold PBS. Cell pellets were fixed by re-suspension in ice-cold 70% ethanol for 30 minutes. Following fixation, cells were incubated with the primary antibodies: phospho (Ser10) histone H3 (D2C8)XP (pH3; Cell Signaling Technology Inc. Danvers, MA, USA, diluted 1:1600). The secondary fluorescent antibody Alexa Fluor 488 (Alexa Fluor goat anti-rabbit; Life Technologies™, Carlsbad, California, USA) was applied 1:500. Cells were washed and further stained with propidium iodide (PI; 1 mg/ml Solution, Life Technologies™, Carlsbad, California, USA) and 10 μg/ml RNase A (Sigma-Aldrich, St. Louis, Missouri, USA). Different cell cycle phases were determinedby flow cytometry using FACSCalibur (Becton Dickinson) by BD Cell Quest Pro software and analyzed with FlowJo 8.7 software. Mitotic cells were characterized by phospho-H3 labeling, while apoptotic cells (sub-G1-phase) were identified by PI staining.

### JC-1 apoptosis assay

This assay determines incipient apoptosis by detection of the initial change in mitochondrial membrane redox potential. Cells were seeded in six-well plates and grown for 48 hours before all cell lines were treated with bortezomib for 16 and 24 hours. Following incubation, cells were harvested and washed in warm PBS. JC-1 staining was performed according to the manufacturer’s instructions. To determine a baseline for positive apoptotic cells, each cell line had a positive control incubated with 2 μl (1:500) of the mitochondrial membrane depolarizer carbonyl cyanide 3-chlorophenylhydrazone (CCCP). CCCP represented 100% of apoptosis in the statistic evaluation. Flow cytometry was conducted using FACSCalibur (Becton Dickinson) by BD Cell Quest Pro software and analyzed with FlowJo 8.7 software.

### RNA interference

Cells were transfected with 40 pmol/ml siRNA in appropriate amounts of Lipofectamine 3000 (Life technologies, Carlsbad, CA, USA) according to the manufacturer’s instructions for 72 h. An endoribonuclease-prepared heterogeneous siRNA pool was used in order to enhance specificity and reduce off-target effects: esiRNA against FOXM1 by Sigma (EHU124431: NCBI reference sequences: NM_021953, NM_202002, NM_202003) or negative control: Sigma Mission^®^ siRNA Universal negative control #1.

### nCounter^®^ multiplex gene expression analysis

Cells were treated in triplets with the substance of interest. RNA was isolated with RNeasy Mini Kit (Qiagen) according to the manufacturer’s instructions. RNA was measured using nanodrop (Thermo Fisher Scientific). 60 ng RNA was applied in the PanCancer^®^ pathway (Nanostring technologies, Seattle, WA, USA) analysis according to the manufacturer’s instruction. Assay was analyzed with nSolver^®^ v2.5 (based on R v3.1.1). The DE results of the gene expression analysis were analyzed with nSolver^®^ v2.5 software (nanostring technologies) using first principal component analysis and regression analysis with and without Benjamini-Yekutieli procedure. The GO overrepresentation analysis was performed with the http://pantherdb.org/ (release 20170413, reference list: Homo sapiens) based on the “panther pathway” annotation data set (PANTHER version 12.0 Released 2017-07-10) with Bonferroni correction for multiple testing [64].

### Animal model and treatment

*In vivo* experiments were carried on the six-week-old Rag2^-/-^yc^-/-^ mice that were purchased from the local stock of the animal facility at Freiburg University. The mice were injected subcutaneously with 1x10^6^ Luc-GFP-H69 cells in 50μl PBS (phosphate-buffered saline) and 50 μl of Matrigel (mouse sarcoma extracellular matrix, Becton Dickinson, Heidelberg, Germany). Bortezomib was administered by intraperitoneal injection in a volume of 0.2 ml at a concentration of 1 mg/kg/day body weight on days 14, 18, 21 and 25 after tumor inoculation. Cisplatin (CDDP; hospital pharmacy, Freiburg) was administered by intraperitoneal injection in a volume of 0.2 ml (5 mg/kg/day) on days 14 and 21 after tumor inoculation. Treatment efficacy was investigated on days 14 and 28 after tumor injection using bioluminescence imaging. Animal protocol was approved by the University Committee on the Use and Care of Laboratory Animals at Albert-Ludwigs University and by the Regierungspraesidium of Freiburg, Germany. When necessary, animals were humanely sacrificed.

### Bioluminescence Imaging

After tumor inoculation presence of human SCLC cells was evaluated via *in vivo* BLI. To quantitatively analyze the progress of viable cells over time, animals were scanned immediately after tumor inoculation and then on week 2, 3 and 5 after tumor engraftment. D-Luciferin was injected intraperitoneally at a dosage of 150 mg/kg. Mice were imaged using IVES CCD (charge-coupled device) imaging system and analyzed using Living Image Software (Caliper Lifesciences).

### Statistical analyses

Statistical analysis of immunohistochemistry was performed with IBM SPSS v22. For univariate analyses containing two dichotomized variables, the χ2 test was applied. For results with low expected counts due to low sample numbers, we applied Fisher’s exact test, if applicable.

*In vitro* data was presented as mean ± standard deviation (SD). We used Tukey’s multiple comparisons test for a two-way ANOVA and multiple T-test to evaluate the significance between different treatment groups of parametric data. To determine whether the values showed a Gaussian distribution, we used the Kolmogorov-Smirnov test. To identify outliers, Grubbs’ method was used. The Mann-Whitney U test was used to compare values between subgroups in order to assess significances of non-parametric data. Statistical significance was defined by p<0.05 (^*^). In the non-parametric test, statistical significance is corrected using the Holm-Sidak method and a definition of alpha=0.05. Analysis was performed with Prism 6 software (GraphPad Software, Inc.).

## SUPPLEMENTARY MATERIALS FIGURES AND TABLES








