# Supplementary material for: Proteasome inhibitor bortezomib enhances the effect of standard chemotherapy in small cell lung cancer
Source: Oncotarget. 2017 Sep 23;8(57):97061–78. doi: 10.18632/oncotarget.21221 (PMC5722545; doi:10.18632/oncotarget.21221)
Supplement: Supplementary file 1 [file oncotarget-08-97061-s001.pdf]

Proteasome inhibitor bortezomib enhances the effect of standard chemotherapy in small cell lung cancer

SUPPLEMENTARY MATERIALS

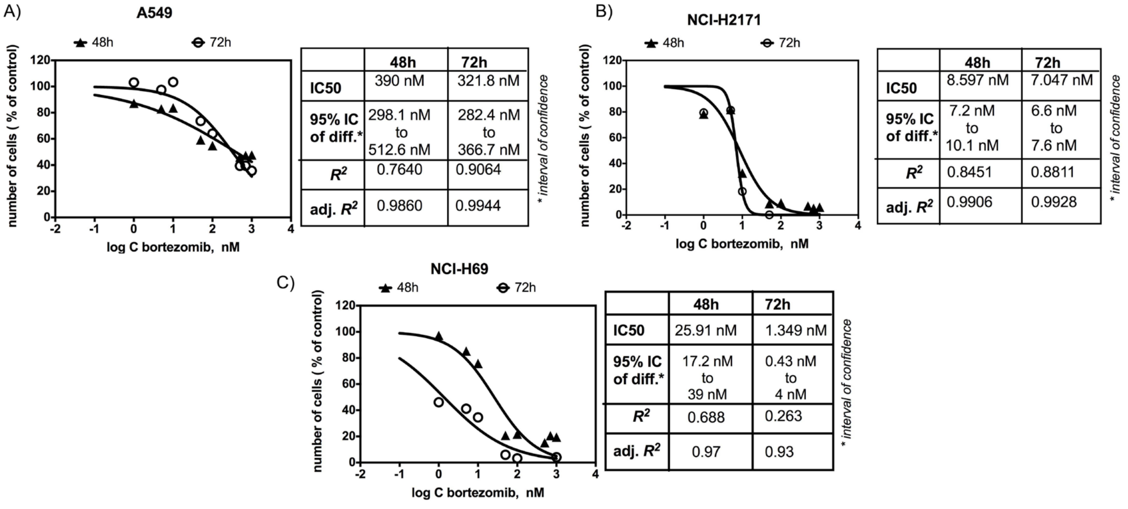

Supplementary Figure 1: Determination of IC50 values of bortezomib.

A)

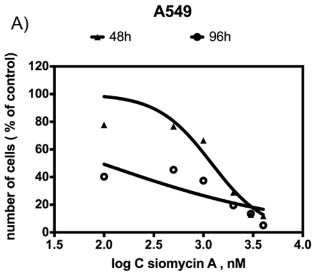

	48h	96h
IC50	1205 nM	93.42 nM
95% IC of diff.*	895.3 nM to 1623 nM	34.54 nM to 252.8 nM
R^2	0.6483	0.4852
adj. R^2	0.9349	0.9081

B)

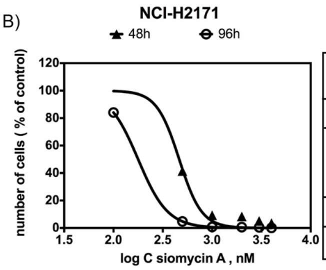

	48h	96h
IC50	464.1 nM	177.9 nM
95% IC of diff.*	370.3 nM to 581.7 nM	166.2 nM to 190.5 nM
R^2	0.8654	0.9947
adj. R^2	0.9760	0.9990

C)

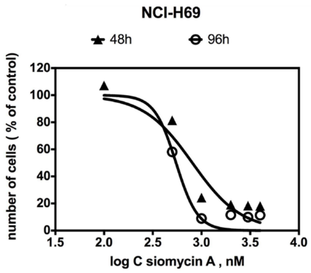

*interval of confidence

	48h	96h
IC50	806.3 nM	548.4 nM
95% IC of diff.*	650.6 nM to 999.2 nM	372.6 nM to 807.1 nM
R^2	0.8724	0.6068
adj. R^2	0.9764	0.9272

*interval of confidence

Supplementary Figure 2: Determination of IC50 values of siomycin A.

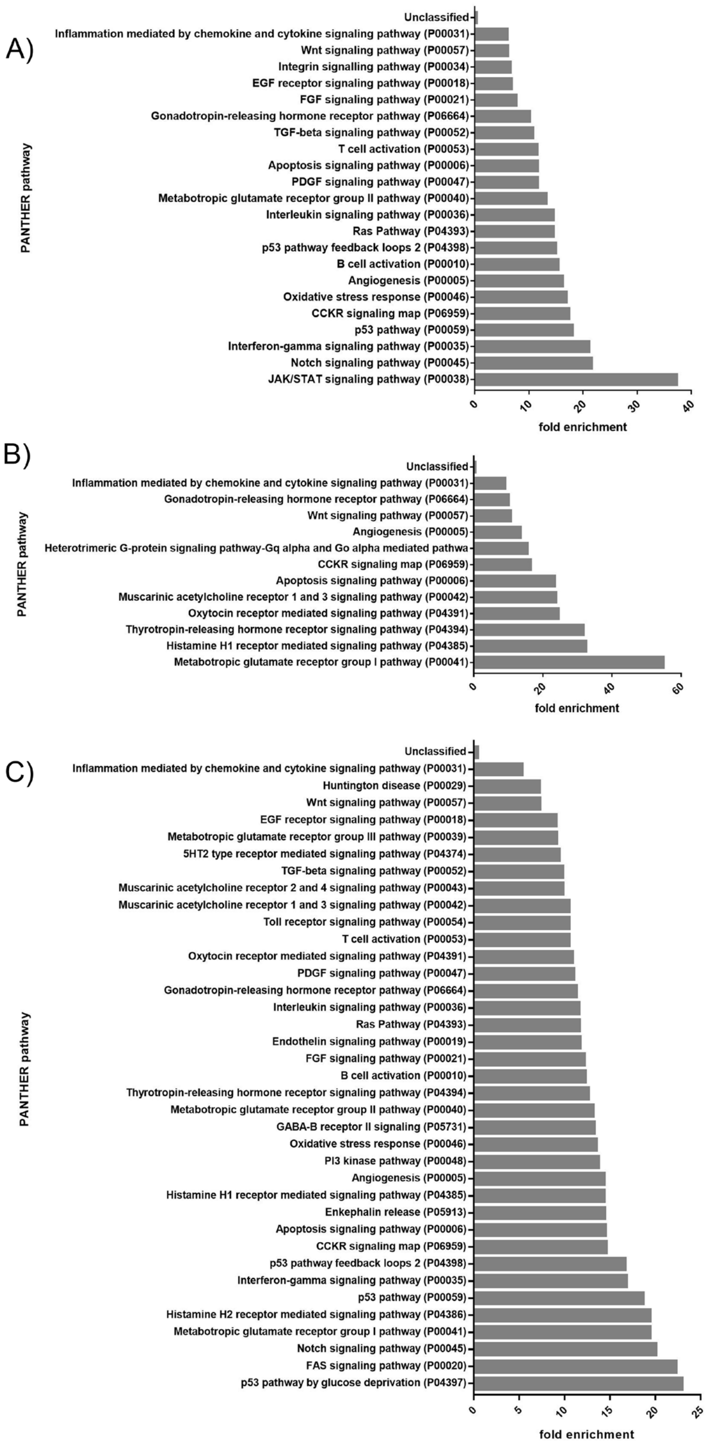

Supplementary Figure 3: PANTHER pathway overrepresentation test of H69 cells treated with 50 nM bortezomib **(A)**, 10 µM cisplatin **(B)** or a combination **(C)** for 48h. Whereas both bortezomib and the combination treatment affected genes of the p53 pathway (such as *GADD45B, DDB2, PML*) to induce an intrinsic apoptotic response (expression of caspase 3 and 7), the combined treatment additionally induced an enhanced extrinsic pro-apoptotic response. This is mediated by activation of caspase 3, 7, 8 and BID expression, as well as a strong inhibition of the anti-apoptotic PI3K pathway by inhibition of AKT and MTOR activation of TSC1 and FOXO4 genes.

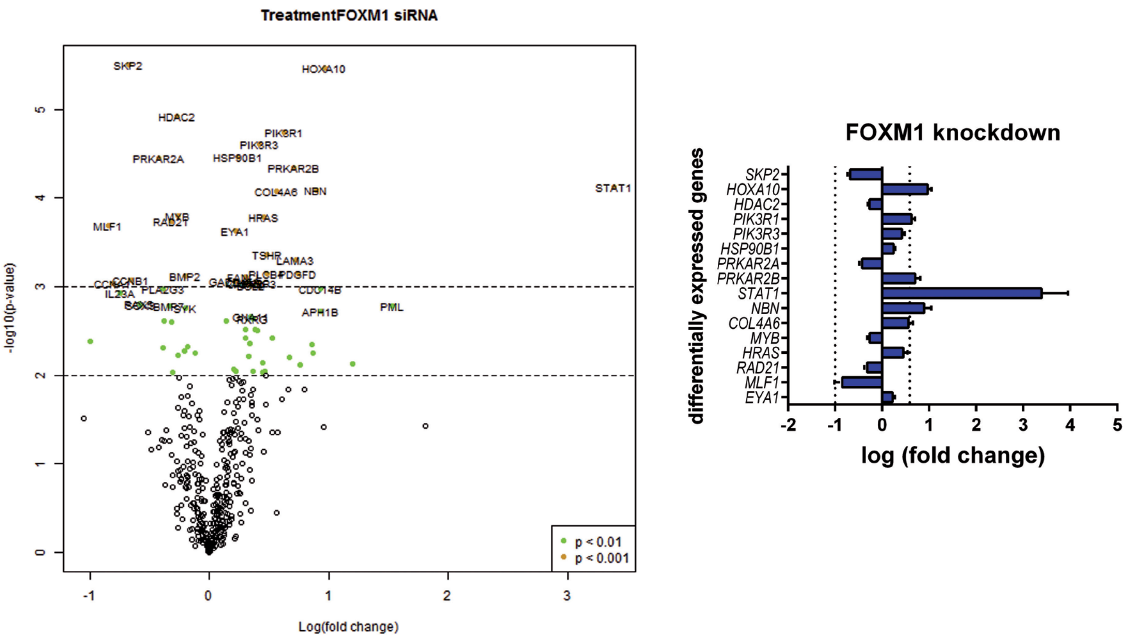

Supplementary Figure 4: Differentially expressed genes after targeting FOXM1 with siRNA. NCI-H69 were transfected with siRNA targeting FOXM1 and analyzed via multiplex gene expression assay (Nanostring technologies). Left: The volcano plot shows differentially expressed genes in respect to log2 (fold change) and significance level (-log10 of p-value). The 40 most significant genes are named. Green dots indicate $p < 0.01$, brown dots indicate $p < 0.001$. Dotted lines mark p=0.01 and p=0.001. Right: The graph shows significantly regulated genes with an FDR < 0.05. Genes are sorted according to significance. Dotted lines indicate a fold change below 0.5 and above 1.5.

Supplementary Table 1: All significantly altered transcripts with a fold change >1.5-fold after bortezomib 50 nM treatment of NCI-H69 cells *in vitro*. Associated Pathways: STAT, PI3K, RAS, MAPK, Wnt, Notch, TGFB, DNA repair, Apop=apoptosis, TXmisReg=transcriptional misregulation, CC=cell cycle, TGFB=TGF-beta, ChromMod= chromatin remodeling, HH=Hedgehog.

See Supplementary File 1

Supplementary Table 2: All significantly altered transcripts with a fold change >1.5-fold after cisplatin 10 µM treatment of NCI-H69 cells *in vitro*

Gene	fold change	P-value	FDR	Pathways
B2M	2,05	2,37E-12	6,71E-09	
BMP7	-1,73	8,16E-07	4,61E-04	TGFB
C19orf40	1,59	2,19E-07	2,06E-04	DNARepair
CACNA1E	-2,12	6,21E-04	3,25E-02	MAPK
CACNA2D1	-1,59	1,07E-03	4,35E-02	MAPK
CACNA2D2	-2,03	6,57E-04	3,31E-02	MAPK
CASP3	1,53	1,13E-04	9,94E-03	MAPK, Apop
CASP7	2,28	1,22E-04	1,01E-02	Apop
CDC25A	1,55	4,78E-05	5,40E-03	CC
CDKN2B	2,61	2,08E-05	3,68E-03	TGFB, CC
CEBPA	2,76	6,50E-04	3,31E-02	TXmisReg
COL4A6	-2,13	1,21E-04	1,01E-02	PI3K
DDIT4	-1,51	3,62E-04	2,28E-02	PI3K
DUSP4	1,69	9,39E-05	8,85E-03	MAPK
EFNA1	3,21	1,56E-05	3,15E-03	PI3K, RAS
FGF14	-1,66	4,40E-04	2,53E-02	MAPK, PI3K, RAS
FOS	4,20	9,43E-04	4,08E-02	MAPK
GNAQ	-1,72	1,49E-04	1,20E-02	
GSK3B	-1,53	2,81E-04	1,89E-02	Wnt, HH, PI3K, CC
HDAC4	-3,17	1,05E-03	4,35E-02	ChromMod
HIST1H3H	-2,40	7,38E-07	4,61E-04	TXmisReg
IL8	11,26	1,28E-05	2,80E-03	TXmisReg
KMT2C	-1,55	1,11E-03	4,35E-02	
MAPT	-1,75	4,69E-05	5,40E-03	MAPK
MNAT1	-1,65	6,72E-04	3,33E-02	DNARepair
NASP	-1,56	1,18E-03	4,49E-02	ChromMod
NF1	-1,61	3,88E-04	2,34E-02	MAPK, RAS
NFKBIA	3,77	8,88E-05	8,69E-03	Apop
PBRM1	-1,63	9,10E-04	4,02E-02	
PDGFD	-2,45	3,30E-05	4,66E-03	PI3K, RAS
PIM1	1,63	4,99E-06	1,57E-03	STAT
PLCB4	-1,86	7,47E-04	3,64E-02	Wnt
PRKCB	-3,29	2,91E-04	1,91E-02	Wnt, MAPK, RAS
RAD51	1,57	8,10E-04	3,81E-02	DNARepair
SOCS1	2,19	6,46E-05	7,02E-03	STAT
SOCS2	1,83	2,58E-04	1,82E-02	STAT
THBS1	4,82	1,74E-05	3,28E-03	TGFB, PI3K
TIAM1	-2,68	3,29E-04	2,12E-02	RAS
TNF	5,76	1,01E-04	9,18E-03	TGFB, MAPK, Apop
TNFAIP3	4,43	2,07E-04	1,58E-02	
TSHR	-1,71	1,27E-03	4,72E-02	
WNT10A	1,86	5,46E-04	2,97E-02	Wnt, HH
WNT11	1,67	8,89E-04	3,99E-02	Wnt, HH
XRCC4	-3,14	1,22E-03	4,61E-02	DNARepair
ZIC2	-1,59	1,16E-05	2,80E-03	HH

Associated Pathways: STAT, PI3K, RAS, MAPK, Wnt, Notch, TGFB, DNA repair, Apop=apoptosis, TXmisReg=transcriptional misregulation, CC=cell cycle, TGFB=TGF-beta, ChromMod= chromatin remodeling, HH=Hedgehog.

Supplementary Table 3: All significantly altered transcripts with a fold change >1.5-fold after bortezomib 50 nM + cisplatin 10 mM treatment of NCI-H69 cells *in vitro*. Associated Pathways: STAT, PI3K, RAS, MAPK, Wnt, Notch, TGFB, DNA repair, Apop=apoptosis, TXmisReg=transcriptional misregulation, CC=cell cycle, TGFB=TGF-beta, ChromMod= chromatin remodeling, HH=Hedgehog.

See Supplementary File 2

Supplementary Table 4: Differentially expressed genes after knockdown of FOXM1 with siRNA. Significant genes ($p < 0.05$) are listed. Genes with an FDR < 0.05 are bold. Change in gene expression is indicated by Log2 (fold change) and the corresponding pathways are listed: Apop = Apoptose, CC = cell cycle, ChromMod = chromatin modification, DNA repair, HH = Hedgehog pathway, MAPK pathway, Notch pathway, PI3K pathway, RAS pathway, STAT pathway, TGFB = TGF-β pathway, TXmisREG = transcriptional misregulation, Wnt pathway.

See Supplementary File 3
