# Supplementary material for: Proteasome inhibitor bortezomib enhances the effect of standard chemotherapy in small cell lung cancer
Source: Oncotarget. 2017 Sep 23;8(57):97061–78. doi: 10.18632/oncotarget.21221 (PMC5722545; doi:10.18632/oncotarget.21221)
Supplement: Supplementary file 2 [file oncotarget-08-97061-s002.pdf]

Supplementary Table 1: All significantly altered transcripts with a fold change >1.5-fold after bortezomib 50nM treatment of NCI-H69 cells *in vitro*

Gene	fold change	P-value	FDR	Pathways
APH1B	-4,18	3,72E-04	1,13E-02	Notch
ARID1A	-1,71	8,22E-04	2,02E-02	ChromMod
ARID1B	-2,42	1,85E-03	3,42E-02	
ARID2	-1,84	1,71E-04	6,77E-03	
ASXL1	2,33	9,35E-04	2,19E-02	
AXIN1	1,98	6,50E-04	1,71E-02	Wnt
BAMBI	1,86	8,94E-04	2,14E-02	Wnt, TGFB
BCL2L1	-2,38	4,43E-06	6,33E-04	TXmisReg, STAT, PI3K, RAS, Apop
BID	1,81	2,80E-04	9,52E-03	Apop
BMP2	1,87	2,02E-04	7,50E-03	HH, TGFB
BMP6	-2,26	1,07E-05	1,26E-03	TGFB
BMP7	-1,59	4,00E-05	2,90E-03	TGFB
BRAF	1,72	4,02E-04	1,20E-02	MAPK
CACNA1E	-2,22	2,10E-03	3,83E-02	MAPK
CACNA2D2	-3,53	2,95E-05	2,38E-03	MAPK
CACNG4	-1,77	1,44E-03	2,90E-02	MAPK
CASP3	1,89	1,96E-05	1,79E-03	MAPK, Apop
CASP7	4,38	3,73E-06	6,33E-04	Apop
CCNA1	-2,15	7,29E-05	4,38E-03	TXmisReg, CC
CCNA2	-2,86	1,06E-04	5,75E-03	CC
CCNB1	-3,80	4,69E-07	2,21E-04	CC
CDC25A	2,04	3,63E-06	6,33E-04	CC
CDC25B	-3,52	3,91E-04	1,18E-02	MAPK, CC
CDC6	1,91	3,66E-06	6,33E-04	CC
CDC7	-2,38	4,33E-06	6,33E-04	CC
CDK2	1,65	1,43E-04	6,14E-03	PI3K, CC
CDKN1A	-1,73	1,07E-05	1,26E-03	TXmisReg, PI3K, CC
CDKN2B	2,82	7,79E-05	4,49E-03	TGFB, CC
CDKN2C	-2,21	2,72E-05	2,26E-03	TXmisReg, CC
CEBPA	9,55	3,15E-06	6,33E-04	TXmisReg
COL2A1	-2,21	2,38E-07	1,52E-04	PI3K
COL4A5	-2,05	3,50E-04	1,11E-02	PI3K
COL4A6	-2,01	1,38E-03	2,83E-02	PI3K
DDB2	-2,84	6,53E-04	1,71E-02	DNARepair
DDIT3	7,35	6,06E-05	3,81E-03	TXmisReg, MAPK
DLL1	-1,60	2,23E-03	3,98E-02	Notch
DLL3	-1,57	2,90E-04	9,58E-03	Notch
DLL4	3,61	1,91E-05	1,79E-03	Notch
DUSP10	3,34	4,48E-05	3,17E-03	MAPK
DUSP4	1,60	1,52E-03	2,99E-02	MAPK
DUSP6	5,31	1,46E-05	1,47E-03	TXmisReg, MAPK
EFNA1	2,45	1,07E-03	2,40E-02	PI3K, RAS
EFNA5	-3,98	3,35E-04	1,07E-02	PI3K, RAS

ENDOG	-2,29	1,38E-03	2,83E-02	Apop
EZH2	1,55	2,41E-03	4,22E-02	
FGF2	2,17	1,14E-03	2,51E-02	MAPK, PI3K, RAS
FOS	25,75	4,21E-06	6,33E-04	MAPK
FOXO4	2,06	8,81E-04	2,13E-02	RAS
GADD45B	21,19	4,79E-05	3,22E-03	MAPK, CC
GADD45G	4,70	2,45E-03	4,22E-02	MAPK, CC
GATA2	3,22	2,12E-04	7,67E-03	
GNG4	-1,73	5,02E-04	1,40E-02	PI3K, RAS
H2AFX	-1,67	2,87E-04	9,58E-03	DNARepair
H3F3A	-1,58	1,18E-07	1,19E-04	TXmisReg
HDAC6	-1,63	1,98E-04	7,46E-03	ChromMod
HES1	1,93	7,97E-04	2,01E-02	Notch
HGF	2,57	1,52E-03	2,99E-02	PI3K, RAS
HIST1H3B	-3,24	1,17E-04	6,13E-03	TXmisReg
HIST1H3G	-6,39	2,62E-05	2,26E-03	TXmisReg
HIST1H3H	-2,50	4,58E-06	6,33E-04	TXmisReg
HSP90B1	1,97	3,62E-04	1,11E-02	PI3K
HSPA1A	64,71	3,57E-04	1,11E-02	MAPK
HSPB1	9,81	2,92E-04	9,58E-03	MAPK
IDH2	-2,53	1,94E-04	7,41E-03	
IKBKG	2,31	5,64E-05	3,62E-03	MAPK, PI3K, RAS, Apop
IL20RA	-1,80	9,86E-04	2,25E-02	STAT
IL23A	1,64	2,42E-03	4,22E-02	STAT
IL8	7,05	6,23E-04	1,68E-02	TXmisReg
ITGA6	-2,08	7,45E-04	1,91E-02	PI3K
ITGB8	1,56	1,26E-03	2,71E-02	PI3K
JAG1	3,17	1,51E-04	6,19E-03	Notch
JAK1	1,75	1,50E-03	2,99E-02	STAT, PI3K
JAK2	2,23	4,48E-04	1,28E-02	STAT, PI3K
JUN	5,56	4,27E-06	6,33E-04	Wnt, MAPK
KLF4	2,60	5,14E-04	1,42E-02	
KMT2D	1,66	1,22E-03	2,66E-02	
MAP2K1	1,69	3,12E-05	2,45E-03	MAPK, PI3K, RAS
MAP3K14	3,05	1,53E-03	2,99E-02	MAPK, Apop
MDC1	-1,80	1,24E-05	1,34E-03	DNARepair
MLF1	2,87	4,64E-05	3,20E-03	TXmisReg
MYC	8,67	4,60E-06	6,33E-04	Wnt, TXmisReg, TGFB, MAPK, STAT, PI3K, CC
MYD88	1,52	1,16E-03	2,54E-02	Apop
NASP	-1,64	2,66E-03	4,52E-02	ChromMod
NR4A1	1,94	1,60E-03	3,06E-02	MAPK, PI3K
NTF3	-2,25	2,16E-04	7,72E-03	MAPK
NTHL1	-1,52	1,42E-05	1,47E-03	DNARepair
PAK3	-1,96	2,24E-03	3,99E-02	RAS
PBRM1	-2,16	1,26E-04	6,14E-03	
PBX3	-1,99	2,33E-04	8,24E-03	TXmisReg
PDGFD	-3,02	3,58E-05	2,66E-03	PI3K, RAS

PIM1	1,75	1,22E-05	1,34E-03	STAT
PML	2,22	9,37E-04	2,19E-02	TXmisReg
POLB	1,90	1,35E-04	6,14E-03	DNARepair
POLR2J	1,87	1,36E-03	2,83E-02	DNARepair
PPP3CA	-1,98	1,81E-03	3,37E-02	Wnt, MAPK, Apop
PPP3CB	-2,35	2,13E-03	3,87E-02	Wnt, MAPK, Apop
PRKACA	-1,72	1,19E-04	6,13E-03	Wnt, HH, MAPK, RAS, Apop
PRKDC	-1,69	9,31E-04	2,19E-02	DNARepair, CC
PRKX	-2,41	9,49E-04	2,20E-02	Wnt, HH, MAPK, RAS, Apop
PROM1	-1,75	1,76E-03	3,29E-02	TXmisReg
PTCH1	-1,81	8,14E-04	2,02E-02	HH
PTPN11	-1,74	7,24E-06	9,30E-04	STAT, RAS
PTTG2	-1,81	8,39E-09	2,37E-05	CC
RAD21	-1,60	1,43E-04	6,14E-03	CC
RASA4	-3,53	4,44E-04	1,28E-02	RAS
RXRG	1,84	1,88E-03	3,45E-02	TXmisReg
SF3B1	1,77	7,26E-05	4,38E-03	
SHC1	1,79	4,21E-04	1,24E-02	RAS
SMAD2	-1,80	1,01E-03	2,28E-02	TGFB, CC
SMAD9	-2,80	8,00E-05	4,52E-03	TGFB
SMARCA4	-2,32	2,06E-04	7,55E-03	
SOCS1	4,59	7,69E-07	3,11E-04	STAT
SOCS3	3,74	1,26E-04	6,14E-03	STAT
SOX9	1,88	2,70E-05	2,26E-03	
STAT3	-1,69	1,46E-04	6,14E-03	STAT
STMN1	-1,79	1,27E-07	1,19E-04	MAPK
SYK	-2,15	1,00E-04	5,56E-03	PI3K
TCF3	-2,30	1,48E-04	6,16E-03	TXmisReg
TFDP1	-1,83	1,35E-04	6,14E-03	TGFB, CC
THBS1	3,54	8,27E-04	2,02E-02	TGFB, PI3K
TIAM1	-2,82	1,26E-03	2,71E-02	RAS
TNC	1,74	2,80E-03	4,72E-02	PI3K
TSHR	-2,11	5,27E-04	1,45E-02	
TTK	-1,71	1,29E-04	6,14E-03	CC
UBB	1,93	2,76E-03	4,66E-02	DNARepair
UBE2T	-2,03	4,70E-06	6,33E-04	DNARepair
WHSC1L1	1,84	1,35E-04	6,14E-03	ChromMod
WNT11	1,84	1,30E-03	2,77E-02	Wnt, HH
WNT6	2,02	1,67E-03	3,16E-02	Wnt, HH
XPA	-1,82	5,18E-05	3,40E-03	DNARepair
ZIC2	-2,08	9,63E-07	3,40E-04	HH

Associated Pathways: STAT, PI3K, RAS, MAPK, Wnt, Notch, TGFB, DNA repair, Apop=apoptosis, TXmisReg=transcriptional misregulation, CC=cell cycle, TGFB=TGF-beta, ChromMod= chromatin remodeling, HH=Hedgehog.
